# High-dose intranasal application of titanium dioxide nanoparticles induces the systemic uptakes and allergic airway inflammation in asthmatic mice

**DOI:** 10.1186/s12931-020-01386-0

**Published:** 2020-07-02

**Authors:** Shaza Abdulnasser Harfoush, Matthias Hannig, Duc Dung Le, Sebastian Heck, Maximilian Leitner, Albert Joachim Omlor, Isabella Tavernaro, Annette Kraegeloh, Ralf Kautenburger, Guido Kickelbick, Andreas Beilhack, Markus Bischoff, Juliane Nguyen, Martina Sester, Robert Bals, Quoc Thai Dinh

**Affiliations:** 1 Department of Experimental Pneumology and Allergology, Faculty of Medicine, Saarland University, Homburg, Germany; 2 Clinic of Operative Dentistry, Periodontology, and Preventive Dentistry, Saarland University, Homburg, Germany; 3 Department of Internal Medicine II, University Hospital, Interdisciplinary Center for Clinical Research Laboratory for Experimental Stem Cell Transplantation, Würzburg, Germany; 4 Department of Internal Medicine, Pneumology, Allergology, and Respiratory Critical Care Medicine, Faculty of Medicine, Saarland University, Homburg, Germany; 5 Leibniz Institute for New Materials, Saarland University, Campus D2 2, D-66123 Saarbrücken, Germany; 6 Institute of Inorganic Solid State Chemistry, Campus Dudweiler, Saarland University, Saarbrücken, Germany; 7 Institute of Medical Microbiology and Hygiene, Saarland University Hospital, Homburg, Germany; 8 Department of Pharmaceutical Sciences, School of Pharmacy and Pharmaceutical Sciences, New York, USA; 9 Transplant and Infection Immunology, Faculty of Medicine, Saarland University, Homburg, Germany

**Keywords:** Asthma, Titanium dioxide nanoparticles, Ovalbumin, Airway inflammation, Hyperresponsiveness, systemic uptake

## Abstract

**Background:**

Titanium dioxide nanoparticles (TiO_2_ NPs) have a wide range of applications in several industrial and biomedical domains. Based on the evidence, the workers exposed to inhaled nanosized TiO_2_ powder are more susceptible to the risks of developing respiratory diseases. Accordingly, this issue has increasingly attracted the researchers’ interest in understanding the consequences of TiO_2_ NPs exposure. Regarding this, the present study was conducted to analyze the local effects of TiO_2_ NPs on allergic airway inflammation and their uptake in a mouse model of ovalbumin (OVA)-induced allergic airway inflammation.

**Methods:**

For the purpose of the study, female BALB/c mice with or without asthma were intranasally administered with TiO_2_ NPs. The mice were subjected to histological assessment, lung function testing, scanning electron microscopy (SEM), inductively coupled plasma mass spectrometry (ICP-MS), and NP uptake measurement. In addition, T helper (Th) 1/Th2 cytokines were evaluated in the lung homogenate using the enzyme-linked immunosorbent assay.

**Results:**

According to the results, the mice receiving OVA alone or OVA plus TiO_2_ NPs showed eosinophilic infiltrates and mucus overproduction in the lung tissues, compared to the controls. Furthermore, a significant elevation was observed in the circulating Th2 cytokines, including interleukin (IL)-4, IL-5, and IL-13 after NP exposure. The TiO_2_ NPs were taken up by alveolar macrophages at different time points. As the results of the SEM and ICP-MS indicated, TiO_2_ NPs were present in most of the organs in both asthmatic and non-asthmatic mice.

**Conclusion:**

Based on the findings of the current study, intranasally or inhalation exposure to high-dose nanosized TiO_2_ particles appears to exacerbate the allergic airway inflammation and lead to systemic uptake in extrapulmonary organs. These results indicate the very important need to investigate the upper limit of intranasally or inhalation exposure to nanosized TiO_2_ particles in occupational and environmental health policy.

## Background

Asthma is a heterogeneous, chronic inflammatory, respiratory disease characterized by recurrent obstructive respiratory events in response to asthma “triggers” [1, 2]. According to the World Health Organization (WHO), annually, hundreds of millions of people suffer from asthma, and over 180,000 people pass away across the world as a result of this condition. Nanotechnology has offered promising strategies for pharmaceutical and therapeutic development by providing such beneficial features as high biodegradability, biocompatibility, adaptability, and minimal toxicity [3–5]. Accordingly, nanoparticles (NPs) have emerged as efficient carriers for several pharmaceutical agents due to their unique physicochemical properties and desirable performance characteristics [6–8].

Clinical trials using NPs have identified some of the reasons underlying acute and chronic diseases and proposed ways to prevent and treat these diseases [9]. The NPs are effective drug transporters given their potentiality to penetrate and remain active in the tissues, cells, and bloodstream [10]. However, the harmful effects of NPs are equally important since they might induce tissue and cellular damage, inflammasome activation, and undesirable modifications [11].

Titanium dioxide (TiO_2_) and its nano-derivatives have a wide range of applications. In this regard, they can be utilized in semiconductors, solar cells, photocatalyst belts, and medicine, as well as in consumer products, including paints, deodorants, toothpastes, sunscreens, and food supplements [12–14]. Nonetheless, it is imperative to also consider the risks induced by TiO_2_ NPs given their harmful effects on the respiratory system and potentiality to augment allergic airway inflammation [15]. This kind of inflammation is transmitted by a complex interplay among different Th2 cytokines, like IL-4, IL-5, and IL-13. The production of cytokines is of significant importance in the pathogenesis of asthma since they further stimulate B cells and eosinophilic inflammation while inhibiting Th1 response [16, 17]. The Th2-mediated allergic asthma and its crosstalk with TiO_2_ NPs suggest the role of underlying cellular machinery in inducing allergic airway inflammation.

In 2017, the European Chemicals Agency (ECHA) Committee for Risk Assessment (RAC) concluded to classify TiO_2_ as a substance suspected of causing cancer through the inhalation route. The RAC classification was made based on the hazardous characteristics of this substance [18]. Regarding this and concerning the growing trend in the production and application of TiO_2_ NPs, there is a rising demand for identifying the consequences of TiO_2_ NP exposure, especially with respect to allergic and inflammatory aspects. Therefore, the present study was conducted to characterize TiO_2_ NPs and investigate their effects on lung tissue morphology, non-pulmonary tissue uptake, in vivo modulation of allergic pulmonary inflammation, and immune response. It was hypothesized that the inhalation of TiO_2_ NPs would have hazardous effects and might aggravate OVA-induced allergic airway inflammation.

## Materials and methods

### Nanoparticles and their physicochemical properties

For the purpose of the study, TiO_2_ NPs (AEROXIDE® P25; Sigma Aldrich, Saint Louis, MO) were utilized with a primary particle size of 21 nm. The measurement of the particles was accomplished by dynamic light scattering (DLS) on a Wyatt DynaPro Plate-Reader II (Wyatt Technology Europe GmbH, Dernbach, Germany) and a Malvern Zetasizer Nano-ZSP (Malvern Instruments GmbH, Herrenberg, Germany) in 96-well plates at room temperature. In this regard, the samples were irradiated with a laser (semiconductor laser with a λ of 830 nm [Wyatt] or a HeNe laser with a λ of 632.8 nm [Malvern]). Subsequently, the intensity fluctuations of the scattered light (detected at a backscattering angle of 156° [Wyatt] or 173° [Malvern]) were analyzed to obtain the autocorrelation function. The device software (Wyatt: DYNAMICS 7.1.9 or Malvern: Zetasizer Software 7.11) outputted the mean particle size using cumulant analysis and a size distribution using a regularization scheme by intensity or number. The mean hydrodynamic diameter was expressed as the log-normal distribution for the intensity or number density, and the dispersity, *p,* was calculated using the following formula:

$$ (p)=\frac{\sigma }{\mu } $$

where *μ* signifies the mean, and *σ* represents standard deviation.

It was assumed that the suspension viscosity was similar to that of water, corrected for temperature. In addition, the suspension refractive index was considered to be equal to that of water (*n* = 1.33). The refractive index of the NPs calculated as 2.4900 with the absorption of 0.01 was applied in the study. The characterization of the NPs was performed using the transmission electron microscopy (TEM). To this end, the NP suspensions were dried at room temperature on pioloform TEM grids and then analyzed with a Tecnai 12 FEI Biotwin TEM setup (Fig. S1).

### Animals

In line with the study objectives, nine-week-old female, wild-type, BALB/c-mice were obtained from the Janvier Labs (Le Genest-Saint-Isle, France) and kept in a 12-h dark/light cycle at 22 °C with laboratory food and tap water ad libitum. The mice were acclimatized for 2 weeks prior to initiating the study. All animal experiments were performed in strict accordance with the German animal protection laws under the approval of the appropriate governmental authority. In addition, every experimental procedure was carried out following the ethical regulations and the animal welfare protocols of the state of Saarland. In order to generate an ovalbumin (OVA) mouse model, the BALB/c mice were intraperitoneally sensitized to OVA (i.e., an allergen), along with aluminum hydroxide-adsorbed OVA (2 mg AlOH_3_ with 20 μg OVA). On the other hand, the control animals received phosphate-buffered saline (PBS) on days 0 and 7. Afterward, the mice were subjected to OVA challenges on days 17, 18, 19, and 20 via the intranasal route (Fig. S2).

In order to prepare the NPs, the treated TiO_2_ NPs were dispersed in double distilled water (Milli-Q®), and the suspensions were ultrasonicated for 15 min to keep the maximum dispersed state. In the NPs groups, each of the BALB/c mice was intranasally treated with 25 μl TiO_2_ NPs suspension (50 mg/mL) 1 h after OVA exposure on days 17 and 20 (Fig. S2). To ensure the homogeneity of the suspension, the stock solutions were vortexed shortly before nasal installation for each mouse. Day 21 was considered the study endpoint. Prior to sacrificing the mice, they were weighed and prepared for pulmonary function testing. Subsequently, the bronchoalveolar lavage fluid (BALF) and some organs were isolated for the implementation of different experiments. Each of the three untreated (i.e., PBS/PBS, OVA/PBS, and OVA/OVA) and three treated groups (i.e., PBS/TiO_2_/PBS, OVA/TiO_2_/PBS, and OVA/TiO_2_/OVA) consisted of 5 mice and 10 mice, respectively.

### Lung function testing

The lung function analysis was performed in our lab and included a non-invasive measurement with conscious animals. Specific airway resistance (sRaw) was performed using a double-chamber head-out plethysmograph (DSI Buxco FinePointe NAM, MN, USA). In addition, the enhancement of doses (0, 12.5, 25, and 50 mg/mL) was accomplished using methacholine (MCh) via an aerosol nebulizer. In this regard, after inserting the mice in the device, they were granted an acclimation period of 5 min to calm down. The aerosol volume was amounted to 0.02 ml and delivered within 1 min. Different MCh concentrations were applied within an interval of 6 min (i.e., 3 min for response time and 3 min for recovery period).

### Tissue sampling and inductively coupled plasma mass spectrometry measurements

After the implementation of airway resistance measurements, the BALB/c-mice were sacrificed by bleeding, and their organs were removed. The lungs were subjected to histological analysis, and BALF analysis was conducted for cell counts. In order to perform ICP-MS screening for titanium (^47^Ti), some portions of the main organs were cut, weighed, and dissolved in 5 mL concentrated ultrapure HNO_3_. Subsequently, a 4% (v/v) solution of ultrapure HCl was added to a final volume of 10 ml. After a few days, the samples were dissolved and analyzed at room temperature using the Agilent 7500cx (Agilent Technologies, Santa Clara, CA). In addition, scandium (^45^Sc) was used as an internal standard.

### Staining and histological analysis

To assess the lung histopathology and airway inflammation, lung cryosections (10 μm) were prepared by means of a cryostat (CM1950, Leica, Germany). Lung tissue cryosections were stained with hematoxylin and eosin (H&E) and periodic acid Schiff (PAS) as previously described [19, 20]. In the next stage, the sections were examined using the Zeiss Axio Imager M2 microscope (Carl Zeiss AG, Oberkochen, Germany). The number of goblet cells in the airways was counted manually after PAS staining under the same light microscope. Furthermore, immunofluorescence (IF) staining was performed with the Shandon Sequenza system (Thermo Scientific, MA, USA). The lung sections of every mouse were dried at room temperature for 15 min. To reduce the nonspecific cross-reactions, the sections were blocked with 5% donkey serum diluted in PBS. Afterward, they were incubated with primary antibodies (i.e., antimouse F4/80 [eBioscience, San Diego, CA], antimouse Ly6G [Abcam, Cambridge, UK], antimouse Siglec-F [eBioscience, San Diego, CA], and antimouse CD3ε [Biolegend, San Diego, CA]) for 1 h at 20 °C and then incubated overnight at 4 °C.

On the second day, the sections were rinsed twice with PBS and then incubated with secondary fluorescein-conjugated antibodies (donkey antirabbit IgG cyanine Cy3, donkey antirat IgG Cy5, and goat anti-Armenian hamster IgG Cy3) for 2 h at room temperature (all secondary antibodies were obtained from Jackson Immunoresearch, West Grove, PA). The cryosections were counterstained with 80 μL 4, 6-diamidino-2-phenylindole (DAPI; 0.5 μg mL^− 1^, Carl Roth, Karlsruhe, Germany) for 15 min, washed several times with PBS and once with double-distilled water, and mounted with Fluoroshield™ fluorescence mounting medium (Sigma-Aldrich, St Louis, MI). Additionally, fluorescence microscopy was performed by means of the Zeiss Axio Imager M2 microscope (Carl Zeiss AG).

### Bronchoalveolar lavage fluid collection

For the collection of BALF, the trachea was exposed by a midline incision in the neck. Subsequently, 1 ml of ice-cold PBS (pH = 7.4) containing protease inhibitors was injected into the lungs through the trachea and withdrawn after 10 s as described previously [21, 22]. In the following stages, the recovered fluid was centrifuged at 1200 rpm for 10 min at 4 °C, the supernatants were removed, and the pellets were resuspended in 0.5 mL PBS. To determine the total cell number, the cells were enumerated by means of a Neubauer cell counting chamber. Afterward, the cytospots were prepared and stained using the Diff-Quick (Medion Diagnostics AG) staining solution in order to discriminate and count the immune cells, including macrophages, neutrophils, eosinophils, and lymphocytes.

### Enzyme-linked immunosorbent assay

After collecting blood samples from the sacrificed animals, they were centrifuged, and the obtained sera were stored at − 80 °C until analysis. Serum concentrations of total immunoglobulin (Ig) E were measured using the commercially available enzyme-linked immunosorbent assay (ELISA) kits (885,046,022, Invitrogen, Vienna, Austria). To determine the protein level in the homogenates, a Pierce BCA protein assay (23,227, ThermoFisher Science, Germany) was performed on the homogenized snap-frozen lungs. After adjusting the protein level in each sample, the ELISA was conducted to analyze the cytokine levels of IL-4 (DY404–05, R&D Systems Inc., USA), IL-5 (DY405–05, R&D Systems Inc., USA), IL-13 (DY413–05, R&D Systems Inc., USA), and interferon-gamma (IFN-γ) (DY485–05, R&D Systems Inc., USA) according to the manufacturer’s protocol.

### Investigation of nanoparticle phagocytosis

The phagocytic ability of primary murine macrophages was analyzed in vitro. To this end, alveolar macrophages were isolated from the BALF of control BALB/c mice (without OVA neither TiO_2_ NPs). The samples were then grown as adherent cultures in RPMI 1640 medium containing 10% FBS and 1% penicillin/streptomycin in eight-chamber culture dishes at 37 °C. The macrophages were treated with 0.0125, 0.025, and 1 mg/mL freshly prepared TiO_2_ NP dispersions for 1, 2, 4, 8, and 24 h. Subsequently, they were fixed with ice-cold acetone and stained with DAPI. Several images were randomly generated using an epifluorescence microscope (Zeiss Axio Imager M2).

### Scanning electron microscopy and energy-dispersive X-ray spectroscopy

The sections obtained from the heart, lung, brain, stomach, kidney, spleen, and liver were scanned for TiO_2_ NPs using an SEM-EDX electron microscope (FEI/Philips XL 30 FEG ESEM; Eindhoven, NL). Moreover, the macrophages in the BALF samples were first stained with Diff-Quick and then scanned for NPs with the same microscope. The tissues, fixed in 2.5% glutardialdehyde and dehydrated in an ascending series of ethanol, dried in 1,1,1,3,3,3-hexamethyldisilazane (Sigma-Aldrich; Taufkirchen, Germany), and coated with carbon, were also evaluated with this setup.

### Statistical analysis

The data were presented as mean ± SEM. Statistical analyses were carried out in GraphPad Prism 5.02 (GraphPad Software, Inc., La Jolla, CA) using one-way ANOVA, followed by Tukey’s test (comparing all pairs of columns). A *p*-value less than 0.05 was considered statistically significant.

## Results

### Nanoparticle characterization

**Fig. 1:**
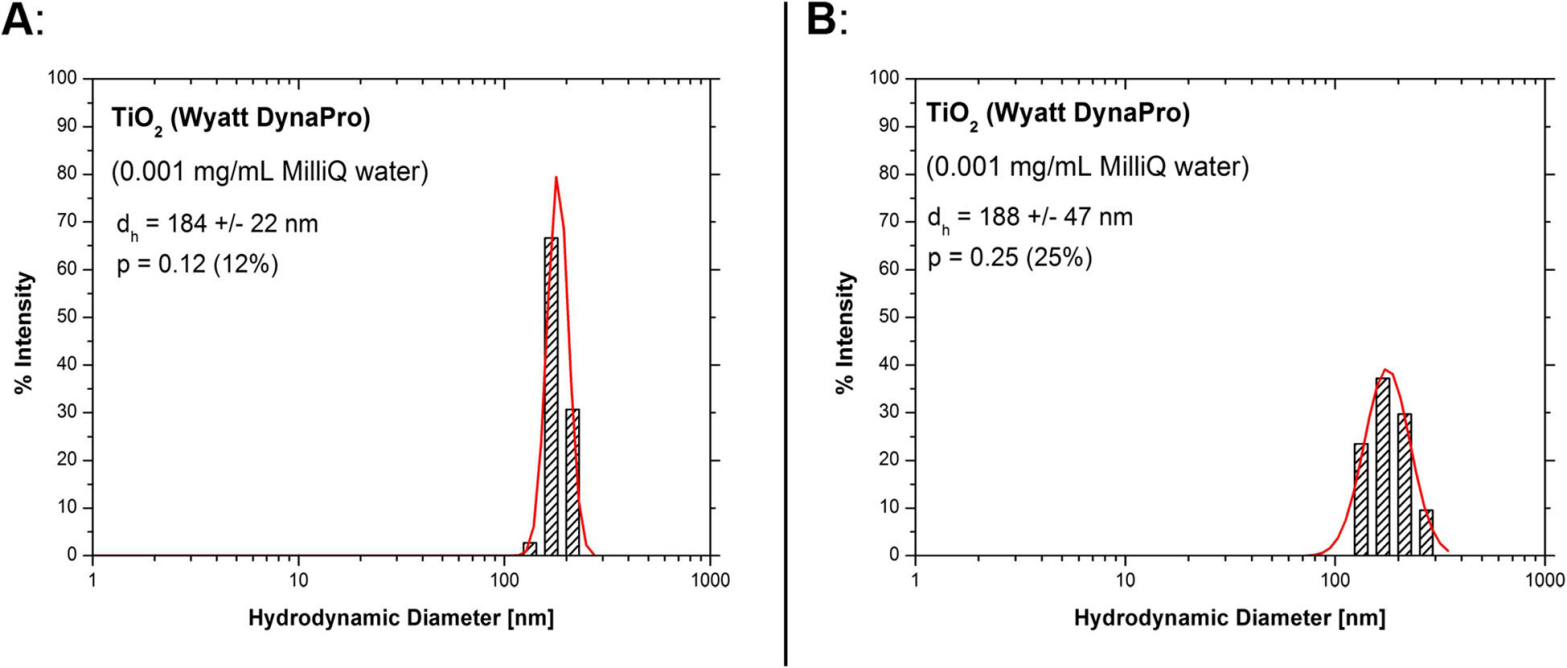
Dynamic light scattering measurements of titanium dioxide nanoparticle suspensions with the maximum measurable concentration (0.001 mg/mL) using the intensity distribution (A comparison was performed between a freshly prepared suspension (**a**) and a suspension prepared 2 h prior to the measurement (**b**). Both samples (**a** and **b**) indicated a monomodal size distribution with a mean hydrodynamic diameter of around 185 nm and dispersity of 12 and 25%, respectively)

The TiO_2_ NPs applied in the current study had a diameter size of 21 nm. The size and distribution of NPs in pure water were determined using DLS. Table 1 presents the physicochemical characterizations of TiO_2_ NPs. The results of DLS analysis demonstrated the monomodal size distribution of both fresh and two-hour-old samples with a mean hydrodynamic diameter of around 185 ± 18 nm. The dispersity of the freshly prepared sample (12%; Fig. 1a) was about half of that of the two-hour-old sample (25%; Fig. 1b), suggesting that the NP suspension becomes unstable and particles start to aggregate/agglomerate over time.

**Table 1:** The physicochemical characterizations of titanium dioxide NPs

Property	TiO_2_
Crystalline structure	Mixture of anatase and rutile with predominantly anatase structure
Primary particle size	21 nm
Hydrodynamic diameter	185 ± 18 nm
Form	Nanopowder
Surface area	35–65 m^2^ /g
Density	4.26 g/mL at 25 °C

### Allergic airways inflammation in mice with intranasally exposure to titanium dioxide particles

The influence of intranasal NP exposure was compared between the non-asthmatic and asthmatic experimental groups. The PBS/PBS and OVA/PBS groups with intraperitoneal OVA sensitization and only PBS challenge, serving as the control groups, showed no allergic airway reaction. On the other hand, the mice in the OVA/OVA groups receiving both intraperitoneal OVA sensitization and challenge, defining the asthma status, developed an asthmatic reaction (Fig. S3A). In the second part, the mice with or without induced airway inflammation were intranasally administered with TiO2 NPs and defined as PBS/TiO2/PBS, OVA/TiO2/PBS, and OVA/TiO2/OVA (Fig. S3B).

**Fig. 2:**
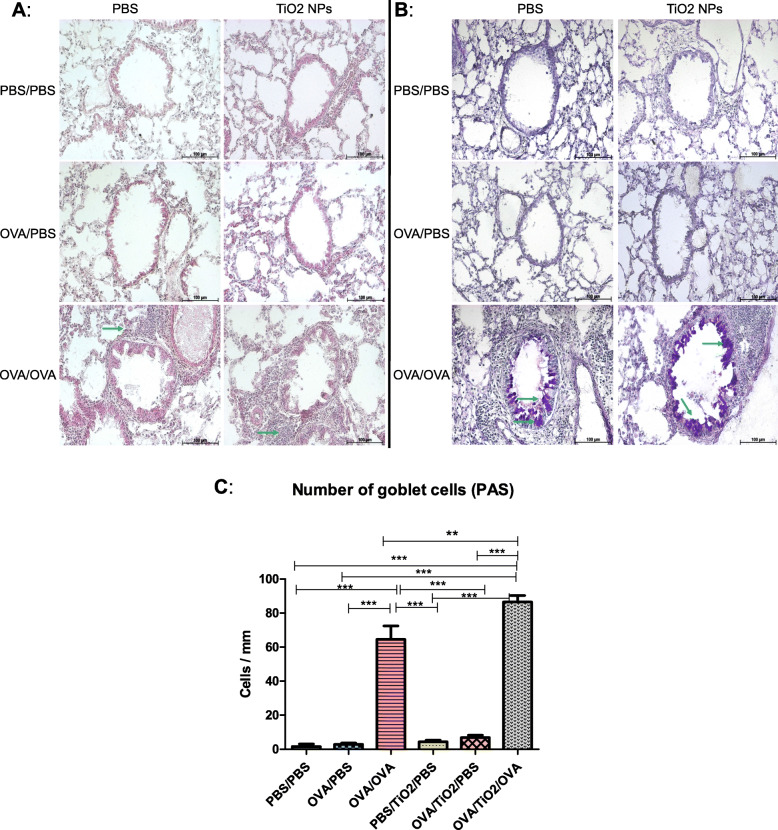
Histological analysis of lung tissue; **a** Representative photomicrographs of fixed lung sections stained with hematoxylin and eosin (Aggregations of inflammatory cells (arrow) were observed in the ovalbumin [OVA]-sensitized and -challenged mice [OVA/OVA] but not in the controls [phosphate buffered saline [PBS]/PBS and OVA/PBS). However, the lungs of the OVA/OVA mice treated with titanium dioxide nanoparticles [TiO_2_ NPs] exhibited more inflammatory cells aggregates), **b** Representative photomicrographs of fixed lung sections stained with periodic acid Schiff (PAS; Lung tissue sections showed goblet cell hyperplasia and increased mucus secretion [arrow] in the OVA/OVA and OVA/TiO_2_/OVA mice, compared to those in the controls), **c** Quantification of goblet cell in PAS-stained lung sections showing a significant increase in goblet cell in the OVA/OVA group, compared to that in the PBS/PBS and OVA/PBS groups (*P* < 0.001; TiO_2_ NPs treatment in the asthmatic OVA/TiO_2_/OVA group resulted in a significant increase in the number of goblet cells, compared to those in the asthmatic non-treated group [OVA/OVA; *P* < 0.01]. Results are expressed as mean ± SEM for seven bronchial airways per mouse [*n* = 5 mice per group], and the groups were compared using one-way ANOVA)

The H&E-stained lung sections obtained from the asthmatic mice (i.e., OVA/OVA) demonstrated a large increase in the inflammatory cells and eosinophilic infiltration, compared to those in the control groups (Fig. 2a). Moreover, these mice showed the hypersecretion of mucus and goblet cell hyperplasia in the PAS-stained sections, compared to the controls (Fig. 2b, c). In contrast, the mice in the non-asthmatic groups (i.e., PBS/PBS and OVA/PBS) had clear airways and less mucus in the respective lung sections (Fig. 2b, c). In half of the animal groups, TiO_2_ NPs were administered in parallel with PBS or OVA. Administration of TiO_2_ to the control groups (i.e., PBS/TiO_2_/PBS and OVA/TiO_2_/PBS) did not cause inflammatory changes, compared to the non-treated groups. However, treatment with TiO_2_ NPs in the OVA-challenged mice with severe asthma (i.e. OVA/TiO_2_/OVA) further increased eosinophilic infiltration, goblet cell counts, and mucus production, compared to those in the asthma untreated group (i.e., OVA/OVA; Fig. 2a-c).

### Enhancement of eosinophil infiltration in the lungs of asthmatic mice as a result of titanium dioxide

**Fig. 3:**
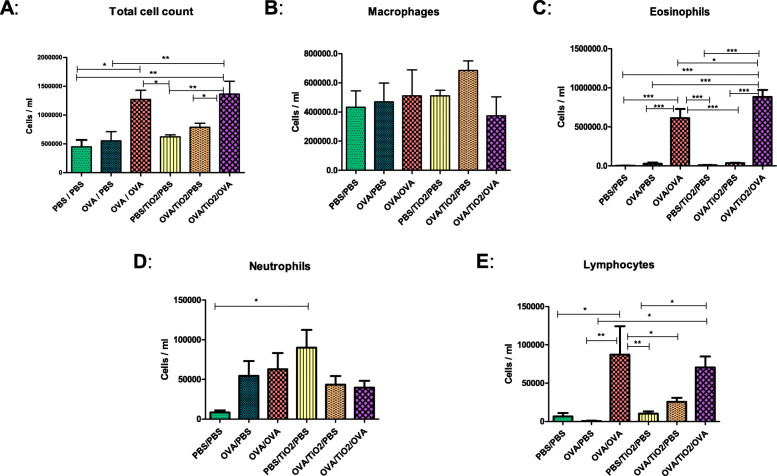
Inflammatory cell count in bronchoalveolar lavage fluid (Total cells (**a**), macrophages (**b**), eosinophils (**c**), neutrophils (**d**), and lymphocytes (**e**) counts in bronchoalveolar lavage fluid obtained from different groups demonstrated an inflammatory response in the lungs after ovalbumin [OVA] exposure. Total cells, eosinophils, and lymphocytes counts were increased significantly in the OVA/OVA mice, compared to those in the phosphate buffered saline [PBS]/PBS controls [*P* < 0.05, *P* < 0.001, and *P* < 0.05, respectively]. Titanium dioxide nanoparticle treatment resulted in a significant increase in eosinophils count in the OVA/OVA mice [*P* < 0.05], indicating a greater asthmatic reaction response. Data are expressed as mean ± SEM for 5 to 10 mice per group. Inter-group comparison was performed using one-way ANOVA)

The immune cell populations present in the BALF of the different experimental groups were also determined in the present research (Fig. 3a-e). The mice in the OVA/OVA asthma group showed a significant increase in total immune cells, compared with the PBS/PBS controls. This increase was exacerbated after TiO_2_ NP exposure (Fig. 3a).

As the results revealed, macrophages were present at almost the same levels in all experimental groups, with an only slight increase in the OVA/OVA mice as compared to those in their non-asthmatic counterparts (Fig. 3b). Furthermore, eosinophils were significantly increased in the asthmatic groups; however, they were almost absent in the controls (Fig. 3c). The TiO_2_ NP mice showed a significant increase in eosinophil counts, compared to all other groups (Fig. 3c). Regarding neutrophils, they were more numerous in the OVA/OVA groups than in the controls. Besides, only PBS/TiO_2_/PBS mice showed a significant increase in the number of neutrophils as compared to the PBS group (Fig. 3d). Lymphocyte numbers, however, were variably different among the groups. In this regard, the OVA/OVA group had a significantly larger number of lymphocytes than the respective non-asthmatic ones (Fig. 3e). Accordingly, it can be concluded that the OVA model was functional and created an eosinophilic inflammatory environment enhanced in the presence of TiO_2_ NPs.

**Fig. 4:**
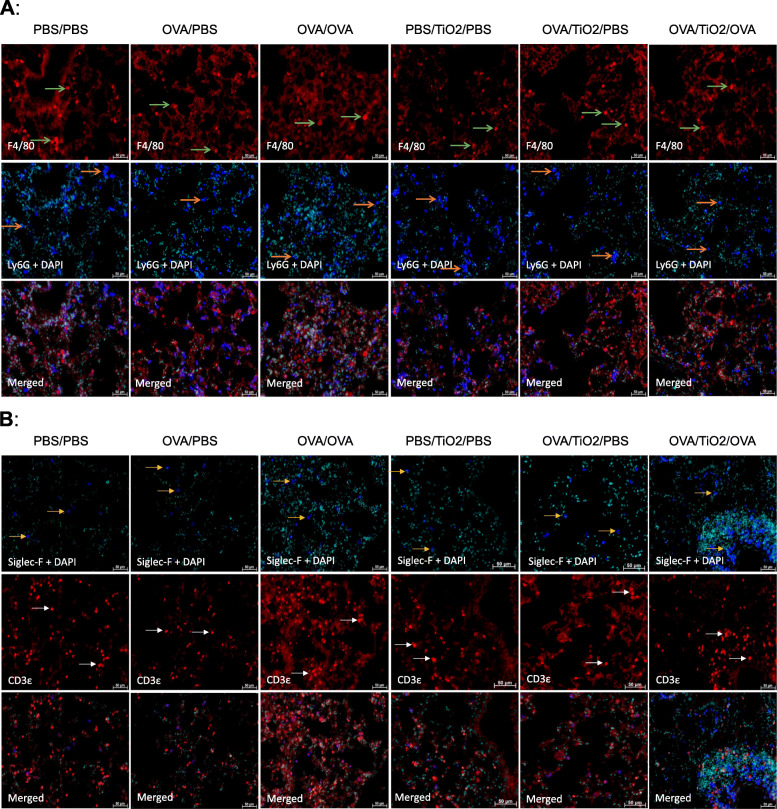
Qualitative immunofluorescence staining of immune cells in the lung sections (Green arrows indicate alveolar macrophages [F4/80 positive, red] and orange arrows represent neutrophils [Ly6G positive, blue]); **a** Eosinophils [siglec-F positive, blue] and T lymphocytes [CD3ε positive, red] as indicated by yellow and white arrows, respectively, **b** Nucleus visualization in cyan using DAPI

To further characterize the inflammatory cell infiltrates in the lung tissues, double IF staining was performed using antibodies directed against macrophage marker F4/80 and neutrophil marker Ly6G (Fig. 4a), as well as eosinophil marker Siglec-F and T lymphocytes marker CD3ε (Fig. 4b). All investigated cells were found in the different tested groups; however, eosinophils and T lymphocytes were prominently present in the OVA/OVA and OVA/TiO_2_/OVA groups than in the controls. This result is in good agreement with the results obtained from the BALF analysis.

### Airway hyperresponsiveness in mice with OVA-sensitization and -challenge with titanium dioxide nanoparticles

**Fig. 5:**
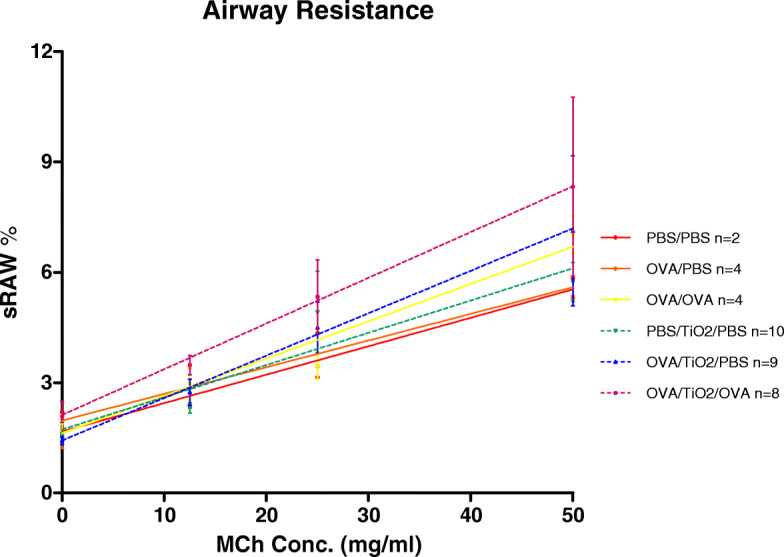
Airway responsiveness to methacholine and percentages of specific airway resistance (sRaw) in the ovalbumin (OVA)-exposed group, titanium dioxide nanoparticle (TiO_2_ NPs)-exposed group, OVA plus TiO_2_ NP-exposed group, and controls upon methacholine stimulation (0, 12.5, 25, and 50 mg/mL) (Mice exposed to OVA showed an increase in sRaw, compared to the controls. Additional exposure to TiO_2_ NPs, along with OVA, resulted in an increase in sRaw. Data are presented as mean ± SEM, and *n* is presented in the graph for each condition)

The pulmonary function test serves to evaluate the severity of airway hyperresponsiveness (AHR). The sRaw is a parameter describing changes in the lung regarding hyperresponsiveness. In order to confirm the effect of OVA-induced asthma on the airways, the changes in the airway resistance were measured upon MCh stimulation. The local effects of NPs in asthmatic mice were evaluated by the measurement of airway resistance in response to the increased doses of inhaled MCh. In line with previous reports [15], our results indicated that mice with severe asthma (i.e., OVA/OVA) showed increased airway resistance in response to MCh in a dose-dependent manner (Fig. 5). In this respect, exposure to TiO_2_ NPs tended to increase the resistance in the PBS/TiO_2_/PBS, OVA/TiO_2_/PBS, and OVA/TiO_2_/OVA groups, compared to that in the untreated mice (Fig. 5).

### Shift of respiratory immune reaction towards a Th2-mediated response as a result of titanium dioxide treatment

**Fig. 6:**
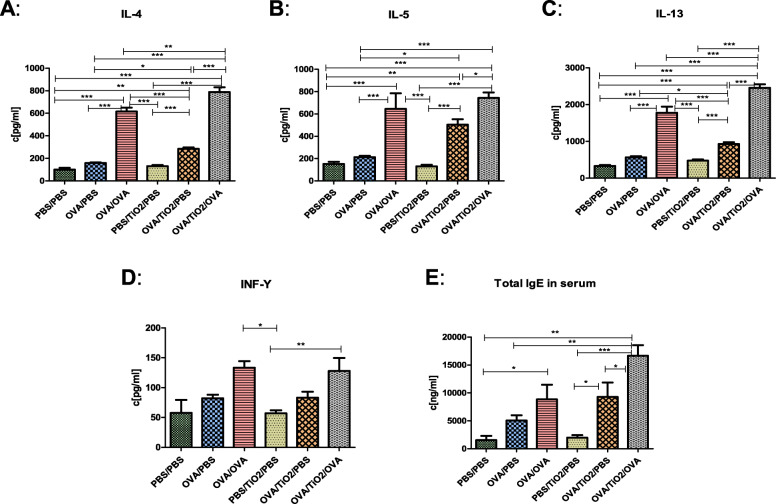
Cytokine measurements in lung homogenates and serum; **a**, **b**, **c** Th2 cytokines (IL-4, IL-5, and IL-13), **d** Th1 pro-inflammatory cytokine (IFN-γ in lungs), and **e** total IgE in serum (Asthmatic mice had an increase in Th2 cytokine levels, compared to the healthy controls. Interleukin levels were higher following ovalubin/titanium dioxide treatment. Data are represented as mean ± SEM [*n* = 5–10], and the groups were compared using one-way ANOVA)

The measurement of Th1/Th2 cytokines revealed significant differences in cytokine levels among the research groups (Fig. 6a-e). In this regard, the OVA/OVA mice showed a significant increase in the IL-4, IL-5, and IL-13 levels in comparison to the non-asthmatic groups (Fig. 6a, b, c). However, the OVA-sensitized and PBS-challenged group had no significant elevation in IL-4, IL-5, and IL-13 as compared to the PBS controls. Furthermore, TiO_2_ NPs were found to significantly increase the IL-4, IL-5 and IL-13 levels in the presence of OVA (OVA/TiO_2_/PBS) (Fig. 6a, b, c). The results also revealed that OVA/OVA combined with TiO_2_ NPs provoked the highest Th2-type cytokine production (IL-4 and IL-13). These findings indicated that treatment with TiO_2_ NPs in the presence of OVA aggravated the asthmatic response; however, this was not observed for particles alone. Although IFN-γ levels remained higher after OVA sensitization and challenge, compared to the PBS controls, TiO_2_ NP treatment exerted no apparent effect on IFN-γ levels (Fig. 6d).

Elevated total serum IgE levels has been established to be associated with allergy. In line with this, circulating IgE levels were significantly increased in the OVA-treated mice (OVA/OVA), compared to those in the non-sensitized group (PBS/PBS). Following TiO_2_ NP exposure, the baseline levels of total IgE were not significantly increased in both OVA/TiO_2_/PBS and OVA/TiO_2_/OVA groups when compared to those in the OVA/PBS and OVA/OVA groups, respectively (Fig. 6e).

### Cellular uptake of titanium dioxide nanoparticles

**Fig. 7:**
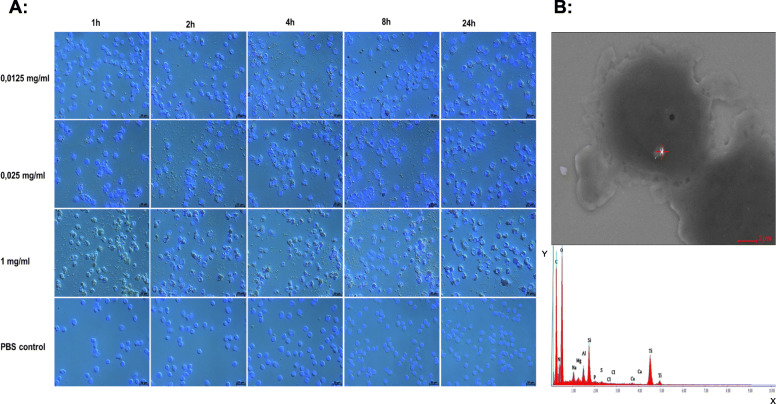
Nanoparticle uptake in alveolar macrophages (Cultured alveolar macrophages (**a**) were exposed to titanium dioxide [TiO_2_ NPs; 0.0125, 0.025, and 1 mg/mL] and phosphate buffered saline. Cellular uptake was analyzed after incubation for 1, 2, 4, 8, and 24 h. Adherent mouse alveolar macrophages were fixed and stained with DAPI before microscopic analysis. An example image of alveolar macrophage in bronchoalveolar lavage fluid (**b**) was taken by scanning electron microscopy. The white spot inside the cell is a cluster of TiO_2_ NPs. This was followed by EDX spectrum analysis showing a strong titanium signal, indicating the presence of TiO_2_ NPs in alveolar macrophages)

To proof whether BALF macrophages take up NPs titanium dioxide nanoparticles as known that the macrophages uptake of NPs by phagocytosis [23]; therefore, the uptake of TiO_2_ NP by isolated BALF macrophages was assessed in the present study (Fig. 7a). Agglomeration mostly occurs prior to phagocytosis, upon the contact of NPs with the cell culture medium. The TiO_2_ NP aggregates were detected in almost all cells at different concentrations and time points. In addition, there were agglomerates precipitated outside the macrophages. The halos and morphological changes of the macrophages seem to be the result of phagocytic activity (Fig. 7a). In order to confirm our previous in vitro results, alveolar macrophages isolated from the BALF of TiO_2_-treated groups were first stained with Diff-Quick and then scanned with SEM, followed by EDX spectroscopy (Fig. 7b). The TiO_2_ NPs could be observed in the alveolar macrophages of the treated mice. This indicated the ability of macrophages to take up TiO_2_ NPs in vivo.

### Titanium dioxide nanoparticle distribution in extrapulmonary organs

**Fig. 8:**
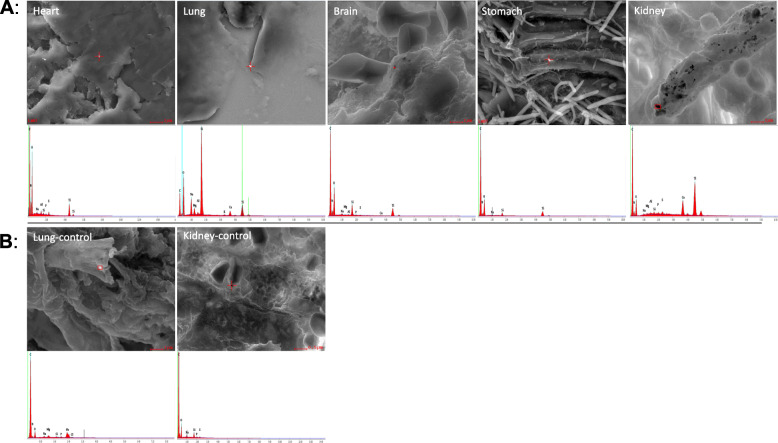
Determination of TiO_2_ NPs in different organs (Images of SEM, followed by EDX spectrum analysis, show the presence of TiO_2_ NPs in the heart, lung, brain, stomach, and kidney of the treated mice (**a**), and the absence of titanium dioxide nanoparticles [TiO_2_ NPs] in the lung and kidney of the untreated mice (**b**))

In order to further examine the distribution and dynamics of intranasally administrated TiO_2_ NPs in vivo, SEM-EDX was performed (Fig. 8a, b). As indicated in the literature, NPs applied via the respiratory tract can cross the blood-air barrier for systemic dissemination [24, 25]. In the present study, the SEM, followed by EDX scans, revealed the presence of TiO_2_ NPs in the heart, lungs, brain, stomach, and kidney samples obtained from the TiO_2_ NPs-treated mice (Fig. 8a). Nonetheless, they were not observed in the untreated mice (Fig. 8b, as examples). To thoroughly analyze the presence of TiO_2_ NPs in more organs, ICP-MS was employed (Fig. S4), revealing titanium traces in the heart, lungs, brain, stomach, kidneys, spleen, and liver (Fig. S4).

## Discussion

Nanosize particles show greater deposition in the alveoli of individuals with asthma and chronic obstructive pulmonary disease (COPD), where they might induce response or exacerbate the disease [26]. The TiO_2_ has recently become part of our everyday lives [27]; accordingly, this compound and its derivative NPs are widely used in technology and medicine [27]. Moreover, TiO_2_ NPs might be found in cosmetics, toothpaste, sunscreens, food supplements, and paints. These particles, when inhaled, have been classified as Group 2B carcinogen by the International Agency for Research on Cancer.

Mishra et al. [15] reported that TiO_2_ NPs increased allergic airway inflammation and Socs3 expression via the NF-kB pathway in a mouse model of asthma. Moreover, Kim et al. [11] demonstrated inflammasome activation in asthmatic lungs after TiO_2_ NP exposure, suggesting the probable contributive effect of targeting the inflammasome on controlling NP-induced airway inflammation. In a human study performed by Heller et al. (2018), the pigment-grade TiO_2_ NPs were reported to be associated with chronic inflammatory degenerative diseases, when inhaled and ingested. The authors also demonstrated that pancreatic TiO_2_ pigment nanocrystals could enter the bloodstream and were associated with type II diabetes mellitus [9].

In line with previous reports, our results indicated that intranasal exposure to TiO_2_ NPs increased the AHR measured during MCh administration in the OVA mouse model. In addition, TiO_2_ NPs were found to enhance eosinophil infiltration in the lungs of asthmatic mice, compared to those in the controls. Eosinophil is well recognized as a major effector cell in the asthmatic airways. The significant elevation of eosinophils is reported to be associated with extreme allergic reactions [28]. Our results also revealed a significant neutrophil influx in the non-allergic mice in comparison to that in the PBS controls.

Neutrophils and their products are the key mediators of the inflammatory changes observed in the airways [29]. This neutrophilic influx is the essential feature of the inflammation reaction induced by TiO_2_ NPs in the PBS control mice, therefore, high-dose TiO_2_ NP inhalation may also have occupational consequences for non-asthmatics.

In addition, TiO_2_ NPs significantly increased the number of goblet cells and consequently mucus secretion in the OVA/TiO_2_/OVA mice, compared to that in the OVA/OVA ones. The significant increase in the serum levels of IL-4 and IL-13 in the OVA/TiO_2_/OVA group was indicative of a strong Th2 response to TiO_2_ NPs exposure. Nonetheless, no alterations were observed in IFN-γ levels; therefore, Th1 was not involved. The OVA-treated mice showed higher total IgE levels following exposure to TiO_2_ NPs; however, this increase was not significant.

Our findings are consistent with the published reports revealing several mechanisms to show the local effects of TiO_2_ NPs on airway inflammation [15, 30–33]. However, to date, limited research has addressed the biodistribution of TiO_2_ NPs into different mice organs after intranasal administration. Therefore, the current study was conducted to describe the systemic uptake of TiO_2_ NPs and their translocation into extrapulmonary organs. Our results indicated that intranasally administered TiO_2_ NPs translocated through the lungs and accumulated in the organs (i.e., liver, spleen, kidney, brain, stomach, and heart) as examined by SEM-EDX and ICP-MS. These particles are small enough to pass through the respiratory tissues into the bloodstream to disseminate into distant organs [34, 35]. In addition, the detection of TiO_2_ NPs in the brain tissue was indicative of the ability of these particles to pass across the blood-brain barrier into the central nervous system following intranasal application, probably via the olfactory bulb by neuronal transport [36, 37].

Moreover, the present study was the first of its kind describing the combination of in vitro and in vivo uptake of TiO_2_ NPs by alveolar macrophages. Based on the evidence, the NPs have the ability to activate phagocytic cells, like macrophages, to take them up. The halos around the macrophages with less NPs precipitation, as well as the macrophage morphological changes, seem to be the result of phagocytic activity [3]. This was supported by in vivo studies (using SEM) showing TiO_2_ NPs inside the BALF-macrophages of NPs-treated mice. Our data also revealed the presence of TiO_2_ NPs aggregates both inside and outside the cells at different time points. This supports the findings of our DLS measurements indicating the instability of TiO_2_ NPs and their aggregation/agglomeration over time.

Generally, the inhalation of nanosized TiO_2_, in combination with OVA, aggravates asthmatic features. The asthmatic exacerbation induced by TiO_2_ NPs is mainly eosinophilic-mediated and enhanced by goblet cell hyperplasia, mucus hypersecretion, increased cytokine levels, and AHR. In view of the findings presented in this study, TiO_2_ NPs alone did not induce strong asthmatic features and inflammatory response in the healthy mice (i.e., non-asthmatics). However, the ability of these particles to translocate into different organs underscores the need for assessing the risk and harmful toxicological potential of these particles in human health. Our findings can provide implications for human health, particularly for asthmatic individuals. Based on our findings, it is recommended that the individuals suffering from asthma or other respiratory diseases (e.g., COPD) limit their exposure to TiO_2_ NPs products. These results carry important implications for occupational and environmental health policy, especially for pre-existing asthmatic populations and workers in industries exposed to TiO_2_ NPs products by various routes.

## Conclusion

The present study involved the examination of the intranasal instillation of TiO_2_ NPs in BALB/c-mice with and without asthma-like airway inflammation. As our data indicated, TiO_2_ NPs did not remain stable over time and agglomerated rapidly; nevertheless, these agglomerates were still observed in alveolar macrophages. The OVA/TiO_2_/OVA mice had high levels of different Th2 cytokines (i.e., IL-4, IL-5, and IL-13). Moreover, intranasal exposure to TiO_2_ NPs in pre-OVA-challenged subjects was found to potentiate the exacerbation of eosinophilic-mediated asthma in the airways. The present study is the first description of pulmonary TiO_2_ NPs uptake by extrapulmonary organs in the context of asthma using SEM-EDX microscopy, followed by ICP-MS measurements.

In light of our findings, it could be concluded that TiO_2_ NPs have an aggravating effect in OVA-challenged mice by modulating the airway microenvironment toward a Th2 immune response. These particles may act as a magnifier of allergic airway diseases, such as asthma. Further investigations are required to better understand the toxicity associated with TiO_2_ NPs accumulation in organs. In addition, more studies are needed to investigate the effects of TiO_2_ NPs on other respiratory diseases.

## Supplementary information

**Additional file 1.**

## Data Availability

The data that support our study are not publicly available due to ethical concerns; however, they can be provided by the corresponding author upon request.

## Abbreviations

TiO_2_: Titanium dioxide
OVA: Ovalbumin
SEM: Scanning electron microscopy
ICP-MS: Inductively coupled plasma mass spectrometry
Th: T helper
IL: Interleukin
WHO: World Health Organization
NPs: Nanoparticles
ECHA: The European Chemicals Agency
RAC: Committee for Risk Assessment
TEM: Transmission electron microscopy
PBS: Phosphate-buffered saline
BALF: Bronchoalveolar lavage fluid
H&E: Hematoxylin and eosin
PAS: Periodic acid Schiff
IF: Immunofluorescence
ELISA: Enzyme-linked immunosorbent assay
Ig: Immunoglobulin
IFN-γ: Interferon-gamma
EDX: Energy-dispersive X-ray spectroscopy
AHR: Airway hyperresponsiveness
sRaw: specific airway resistance
MCh: Methacholine
COPD: Chronic obstructive pulmonary disease
