# Supplementary material for: High-dose intranasal application of titanium dioxide nanoparticles induces the systemic uptakes and allergic airway inflammation in asthmatic mice
Source: Respir Res. 2020 Jul 2;21:168. doi: 10.1186/s12931-020-01386-0 (PMC7331175; doi:10.1186/s12931-020-01386-0)
Supplement: Supplementary file 1 — Additional file 1. [file 12931_2020_1386_MOESM1_ESM.doc]

**Supplementary Information**

High-dose intranasal application of titanium dioxide nanoparticles induces the systemic uptakes and allergic airway inflammation in asthmatic mice

Shaza Abdulnasser Harfoush^1^, Matthias Hannig^2^, Duc Dung Le^3^, Sebastian Heck^1^, Maximilian Leitner^1^, Albert J. Omlor^1,7^, Isabella Tavernaro^4^, Annette Kraegeloh^4^, Ralf Kautenburger^5^, Guido Kickelbick^5^, Andreas Beilhack^3^, Markus Bischoff ^6^, Juliane Nguyen^7^, Martina Sester^8^, Robert Bals^9^and Quoc Thai Dinh^1,9^


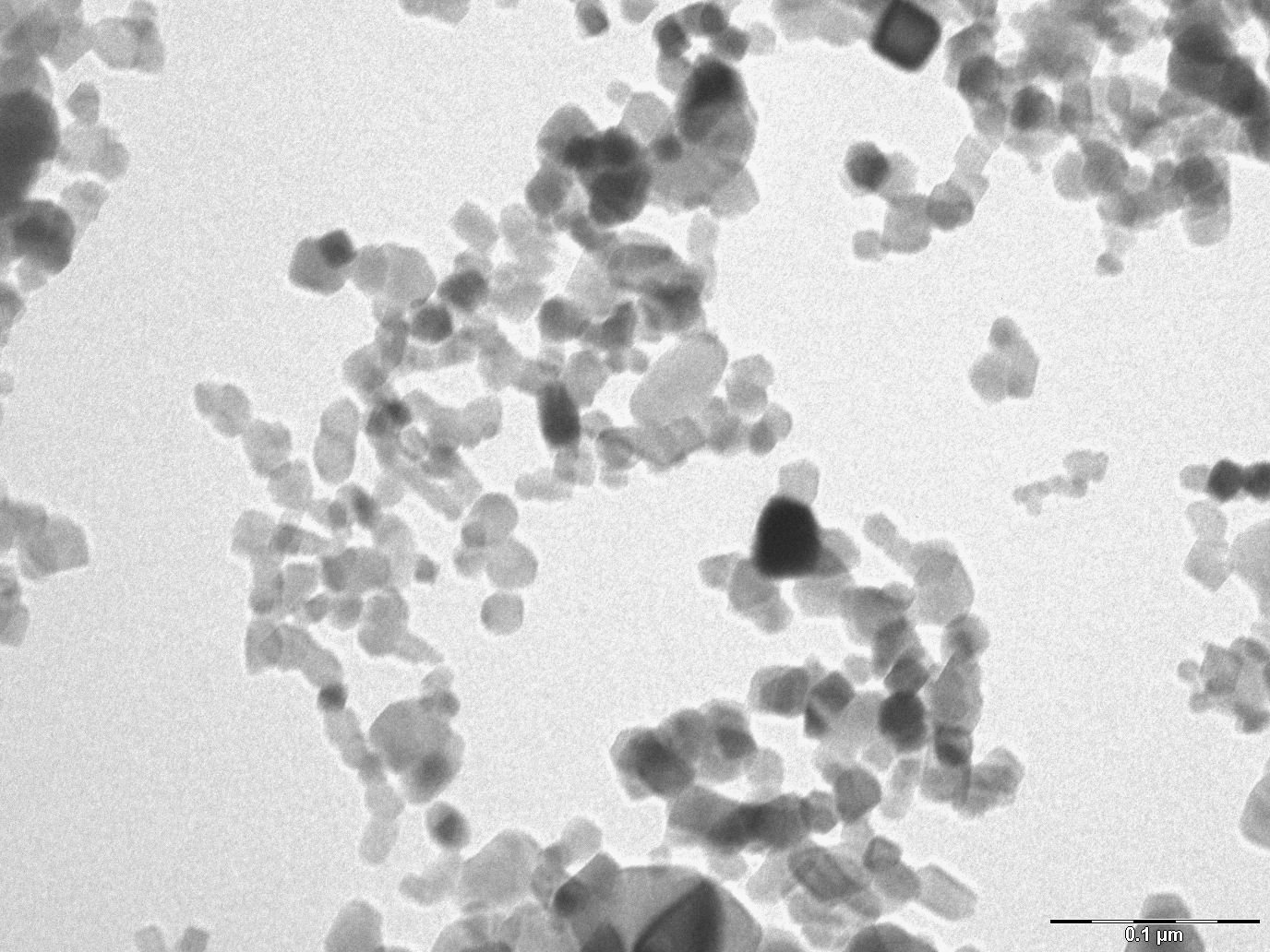


**Fig. S1.** Transmission electron microscopy image of titanium dioxide nanoparticles (TiO_2_ NPs; The NPs had a core diameter of approximately 21 nm. Results revealed that the majority of TiO_2_ NPs were spherical and tended to create relatively soft-bonded agglomerates.)


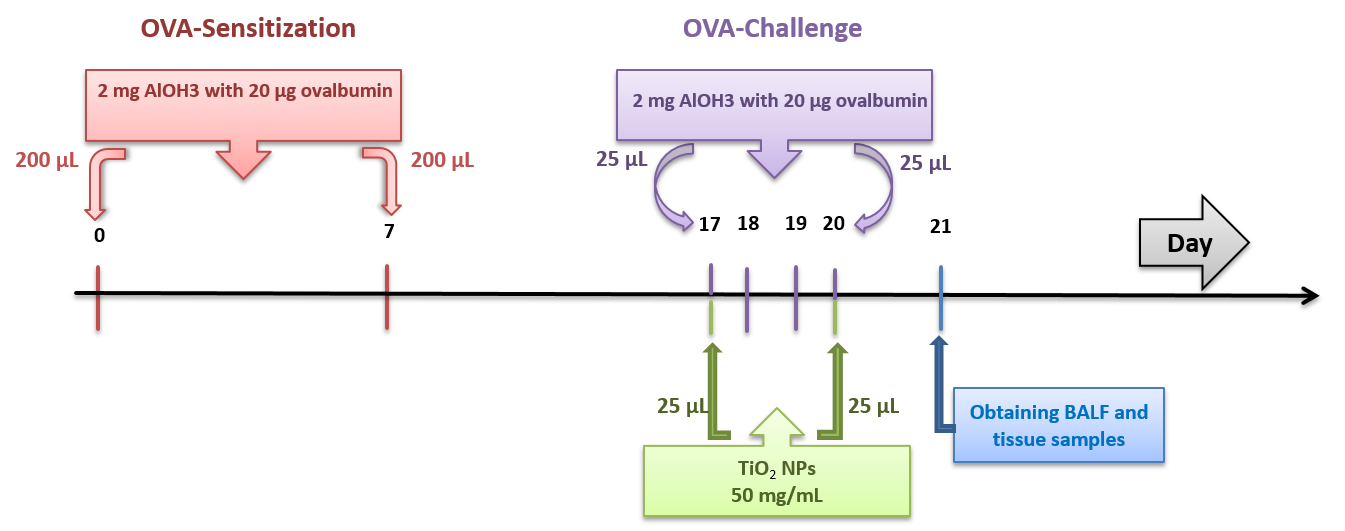


**Fig. S2.** Sensitization and challenge procedures in the mice.

Mice in the control groups received an intraperitoneal injection of 200 µl PBS or aluminum hydroxide-adsorbed ovalbumin (OVA; 2 mg AlOH_3_ with 20 µg OVA) on days 0 and 7. On days 17, 18, 19, and 20, the mice received intranasally 25 µl OVA for OVA challenge. In the NPs groups, each of the BALB/c mice was treated intranasally with 25 µl TiO_2_ NPs suspension (50 mg/mL) 1 h after OVA exposure on days 17 and 20. Day 21 was defined as the final study endpoint. Airway resistance measurements were monitored in terms of methacholine-induced specific airway resistance. The bronchoalveolar lavage fluid was collected for cell counting, and tissue samples were isolated for histologic analysis.


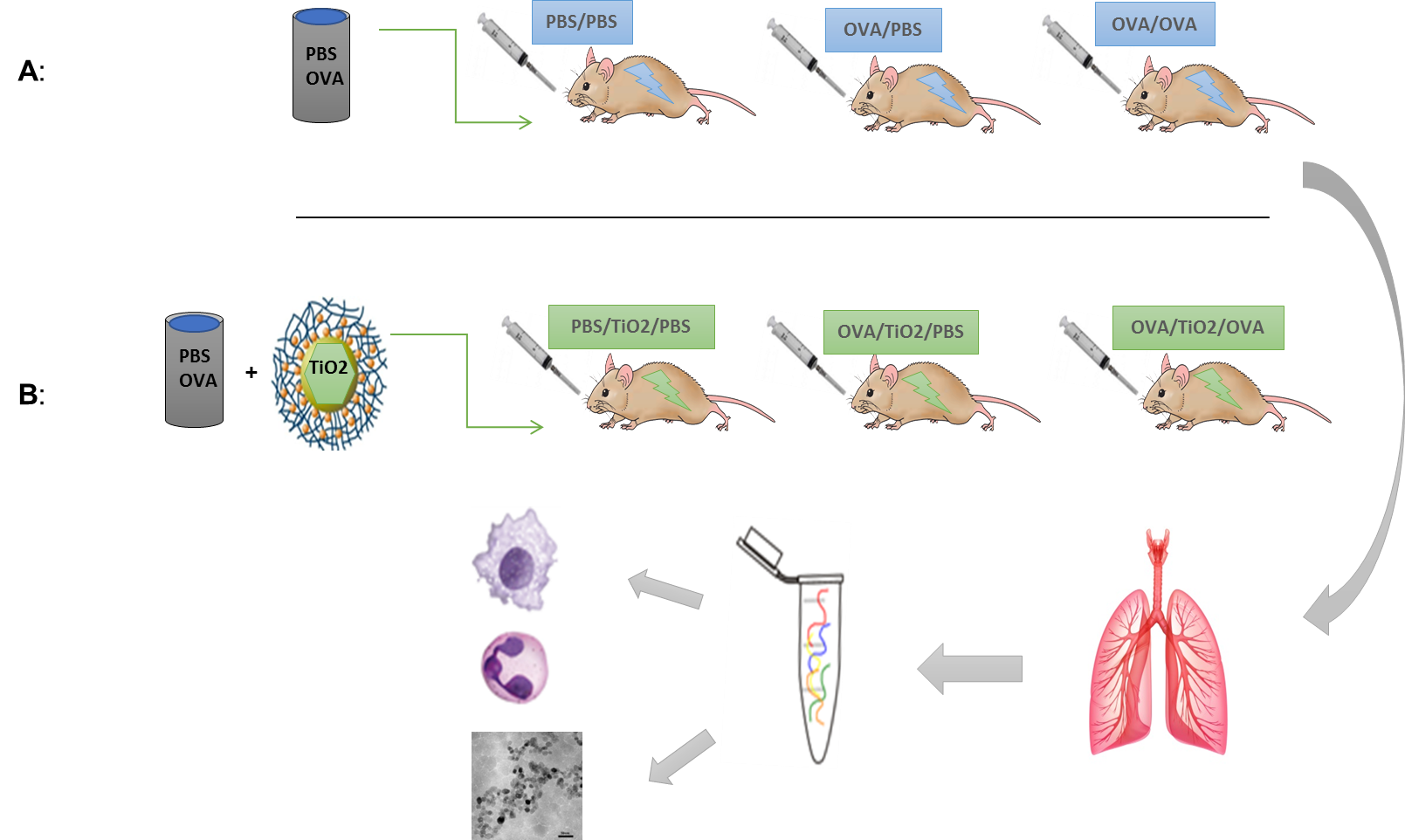


**Fig. S3.** Investigation of BALB/c female (9-week-old) mice in six test groups.

Mice in the phosphate-buffered saline [PBS]/PBS group were neither sensitized nor challenged and therefore had no asthma [**A**]. However, those in the ovalbumin [OVA]/PBS group were sensitized to OVA but not challenged and therefore had no asthma [**A**]. The first two categories together define the non-asthmatic or control status. Mice in the third group with OVA/OVA were both sensitized and challenged with OVA and therefore had asthmatic reactions [**A**]. In [**B**], we applied titanium dioxide nanoparticles [TiO_2_ NPs] in combination with the previous treatment to the mice. Three new groups receiving NPs were defined as PBS/TiO_2_/PBS, OVA/TiO_2_/PBS, and OVA/TiO_2_/OVA [**B**]. Each of the first three groups consisted of 5 mice. Each of the second three groups was composed of 10 mice.


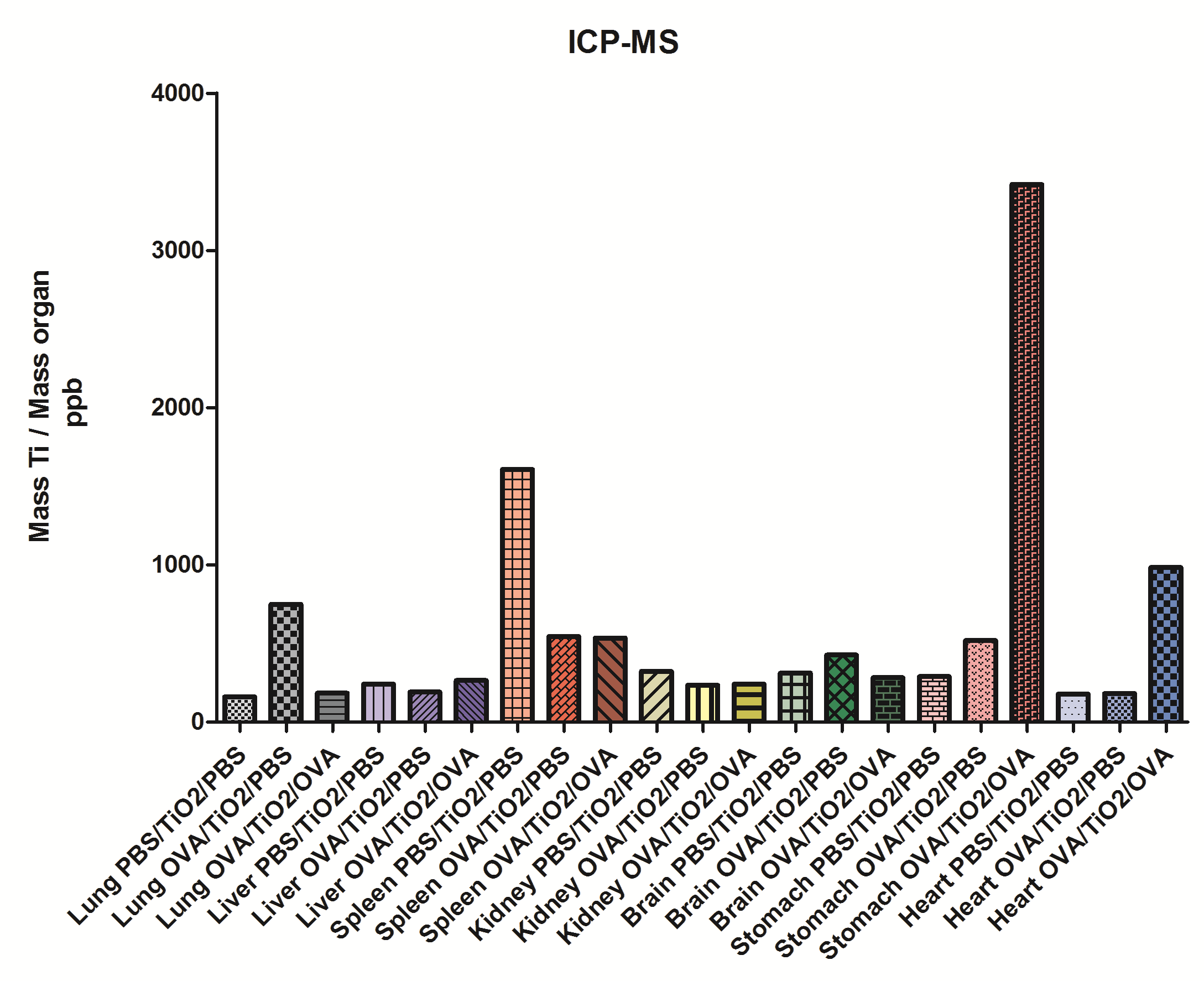


**Fig. S4.** Inductively coupled plasma mass spectrometry of different organs showed the traces of TiO_2_ NP in the heart, lung, brain, stomach, and kidney of the treated mice expressed as mass Ti/mass organ in ppb.
